# Structural and morphological peculiarities of hybrid Au/nanodiamond engineered nanostructures

**DOI:** 10.1038/srep31163

**Published:** 2016-08-12

**Authors:** Roberto Matassa, Silvia Orlanducci, Giacomo Reina, Maria Cristina Cassani, Daniele Passeri, Maria Letizia Terranova, Marco Rossi

**Affiliations:** 1 Department of Basic and Applied Sciences for Engineering, Sapienza University of Rome, Via Antonio Scarpa 16, 00161 Rome, Italy; 2 Department of Chemical Science and Technology-MINIMAlab, University of Rome “Tor Vergata”, Via della Ricerca Scientifica, 00133 Rome, Italy; 3 Department of Industrial Chemistry ‘Toso Montanari’, University of Bologna, Viale del Risorgimento 4, 40136 Bologna, Italy; 4 Center for Nanotechnology for Engineering (CNIS), Sapienza University of Rome, P. le A. Moro 5, 00185 Rome, Italy

## Abstract

Nanostructured Au nano-platelets have been synthesized from an Au(III) complex by growth process triggered by nanodiamond (ND). An electroless synthetic route has been used to obtain 2D Au/ND architectures, where individual nanodiamond particles are intimately embedded into face-centered cubic Au platelets. The combined use of high resolution transmission electron microscopy (HR-TEM) and selected area electron diffraction (SAED), was able to reveal the unusual organization of these hybrid nanoparticles, ascertaining the existence of preferential crystallographic orientations for both nanocrystalline species and highlighting their mutual locations. Detailed information on the sample microstructure have been gathered by fast Fourier transform (FFT) and inverse fast Fourier transform (IFFT) of HR-TEM images, allowing us to figure out the role of Au defects, able to anchor ND crystallites and to provide specific sites for heteroepitaxial Au growth. Aggregates constituted by coupled ND and Au, represent interesting systems conjugating the best optoelectronics and plasmonics properties of the two different materials. In order to promote realistically the applications of such outstanding Au/ND materials, the cooperative mechanisms at the basis of material synthesis and their influence on the details of the hybrid nanostructures have to be deeply understood.

Nowadays, hybrid nanocrystalline particles, at the basis of many advanced materials for frontier applications in several technological fields ranging from optoelectronics to nanocatalysis and to biomedicine, receive an increasing attention owing to their outstanding properties^1^,^2^. In this context, a key point is represented by the design of hybrid nanostructures and of their production routes. To this purpose, the definition of production protocols, able to control the growth process and innovative crystallization pathways is a primary issue.

In fact, in the case of metal/nonmetal hybrid structures, which can be synthesized by both epitaxial^3^ and non-epitaxial routes^4^,^5^,^6^, outstanding properties are often related to the coupling of different nano-crystals since a suitable microstructure could induce a synergic behavior able to enhance the intrinsic chemical-physical properties of each constituent.

However, being the properties strongly related to the microstructure, an essential requirement for the production of nano-hybrids with enhanced properties is to master the synthetic route in order to control the modulation of the whole structure, mainly as far as uniformity of size, shape, composition, and structure of interfaces are concerned.

Several studies have been recently dedicated to the atomic scale characterization of noble metal/Carbon nanomaterials. In fact, these hybrid systems are indeed very promising for application in nanophotonics/nanoelectronics as well as in nanomedicine/nano-biochemistry^7^,^8^,^9^,^10^,^11^,^12^,^13^,^14^,^15^,^16^. Moreover, a synergic approach can offer interesting solutions to technological problems related to optical labeling, imaging, molecular loading and biosensing.

Among the various nanosized forms of Carbon allotropes, nanodiamond (ND)– i.e., diamond nanoparticles 4–5 nm in size – is deeply studied in relation to different applications owing to its unique properties^9^,^17^,^18^. In fact, the complex surface structure allows a great variety of chemical functionalizations without compromising the outstanding functional properties of the ND core^19^. In the last years, particular interest has been attracted by diamond containing color centers, which have been proposed for a series of applications, not only in photonics and optoelectronics, but also in modern medical and biological research^20^,^21^,^22^,^23^,^24^.

On the other hand, among the noble metals nanostructures, Au nanoparticles are of particular interest due to the peculiar and outstanding chemical-physical properties, which are strictly depending on size, shape and details of the crystal structure.

Au nanoparticles generally show a high density of defects at the surface^25^,^26^; in fact, during the growth process, the creation of such defects in the seed crystal is energetically favored, promoting a preferential diffusion path for Au atoms to be incorporate at the surface^27^.

In the case of single crystalline seeds, surface defects can act as nucleation sites facilitating secondary growth and the creation of new interfaces and grain boundaries, which, in turn, drive the evolution of the shape of Au nanoparticles. These features enabled the manufacturing of Au nanoparticles into a variety of shapes including nanospheres, nanotriangles, nanorods, nanobelts, nanocages, nanoprisms, and nanostars^28^. On the other hand, the availability of variegated morphologies of Au nanoparticles, stimulated the exploration of different applications, e.g., in the emerging field of plasmonics^29^ and related theranostic applications^30^. In fact, Au nanoparticles are widely investigated as active components in systems for focusing and guiding visible light and in field-enhanced phenomena.

On this basis, the engineering of Au/ND nanosystems presents interesting prospective. In particular, the synergy between the two components may result in a significant enhancement of the plasmonic emission properties, with far-reaching implications for several forefront applications.

As an example in such a context, coupling Au with NDs containing color centers, results in an increase of the photonic yields of two order of magnitude even at room temperature and without any photodegradation process^31^,^32^. The optical properties of hybrid Au/ND systems can be tailored to fit an enormous range of possible applications^31^,^32^,^33^, provided the capability of controlling not only size and shape of the crystallites, but also the mutual organization and the structure of the interphase interfaces.

However, the availability of reliable synthetic methods for the tailored production of Au/ND nanostructures still represents the main bottleneck for fully exploiting the multifunctional properties expected for this exciting two-phase system.

Recently, some of the present authors^34^ proposed a new, simple and eco-friendly method to synthesize Au/ND nanostructures, based on the direct reduction of an Au(III) complex on nanodiamond surface. Preliminary results encouraged us to extend this chemical route for the realization of more complex Au/ND nanosystems.

In this work, we present an accurate investigation at the nanoscale of Au/ND hybrid materials grown by the abovementioned synthetic route. In particular, we focused the investigation on the first stage of the growth process with the purpose of better describing the basics of the synthesis mechanism. To investigate the particle microstructure at atomic scale we used transmission electron microscopy (TEM) coupled with selected area electron diffraction (SAED). Energy dispersive X-ray spectrometry (EDX) confirmed the formation of pure metallic Au, while high resolution TEM (HR-TEM) provided the details of the microstructure. Moreover the analysis of HR-TEM image by Fast Fourier Transform (FFT) and Inverse Fast Fourier Transform (IFFT) allowed ascertaining the mutual relationship between nanocrystals of different nature.

## Experimental

Detonation nanodiamonds (NDs)^9^ with 4–5 nm primary particles size and 30 nm average agglomerate size (ITC standard Nanodiamond, purity >98%) was further purified and disaggregated using chemical-physical procedures^35^ to generate a high density of OH groups at the surface^9^,^36^ and finally dispersed in water by sonication up to a final concentration of 0.1%w.

Au(III)-aminoethyl imidazolium cationic complex [Cl_3_AuNH_2_(CH_2_)_2_imMe][AuCl_4_] was synthesized according to the procedure reported in ref. 37. A 7·10^−4^ M solution in water was then prepared without the use of any supplementary additive and/or stabilizer.

Au/ND particles were produced by an electroless chemical route set up in our labs. In particular, we followed the synthesis route 1 described in ref. 34, where 30 mL of ND dispersion are mixed with 90 mL of Au complex solution, with the pH maintained at 6.5. The resulting mixture, kept under moderate agitation, became light-pink in about 5 minutes, indicating the precipitation of Au nanoparticles. The reaction in the solution proceeds up to 10 days as evidenced by the color evolution. After 10 days in fact, the solution showed an orange-brown color. Au/ND particles were collected by several centrifugations and washing cycles in order to eliminate salts and unreacted species. The samples analyzed in this work were collected 15 minutes after the onset of the reaction.

TEM images and electron diffraction patterns were collected with a JEOL2010 @200 kV while HR-TEM images and EDX analysis were obtained with a JEOL3000F @300 kV. Raman spectra were acquired using an *eXplora* system (Horiba) with a 532 nm laser source, a power less than 1 mW, a 100x magnification and 3 cm^−1^ spectral resolution.

## Results and Discussion

A preliminary morphological and structural analysis was carried out on the ND powder to assess nanoscale features before the reaction with Au, and in particular, to verify the efficiency of the adopted protocol in removing graphitic-carbon impurities and promoting the formation of hydroxyl (alcoholic and/or enolic) groups at the surface which represent the sites for Au^+3^ reduction.

**Figure 1:**
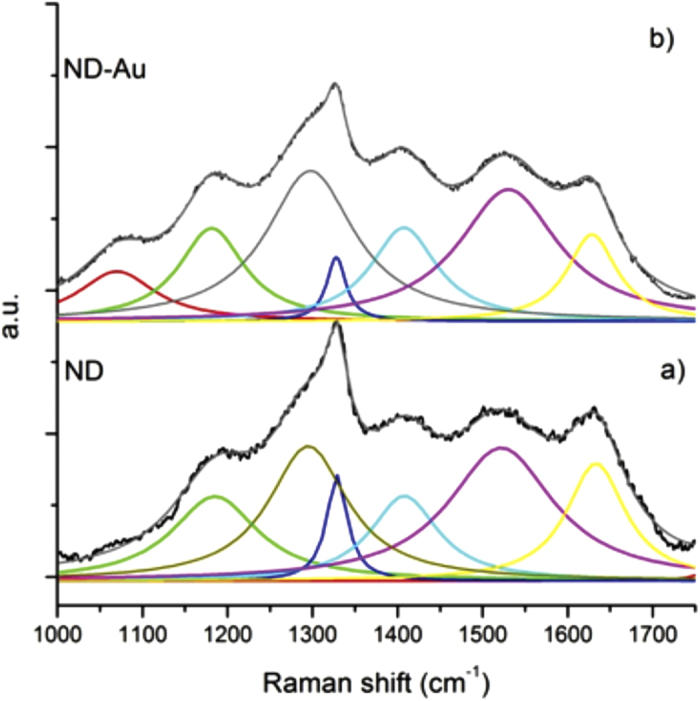
Normalized Raman spectrum of purified ND (**a**); and ND-Au system (**b**). The fit has been carried out by using a multi Lorentzian function.

The Raman spectrum of purified ND, reported in Fig. 1a, exhibits the typical Raman profile of detonation nanodiamond, with a relevant band attributed to nanodiamond lattice positioned at 1320 cm^−1 ^38,39. A full spectral analysis comprises also the following components: a band positioned at 1520 cm^−1^ ascribed to G-bands of disordered sp^2^ carbon species, the bands at 1180 and 1410 cm^−1^ attributed to transpolyacetilene forms^40^ and spectral features at 1630 cm^−1^ assigned to surface OH groups^41^, produced at the surface of detonation ND by the purification and dispersion process^42^,^43^. It is possible to notice that the graphitic phases are present in very low concentration, considering that the Raman efficiency for diamond is much smaller than for graphite^44^.

The spectra relative to purified ND (Fig. 1a) and Au/ND (Fig. 1b) samples show a very similar bands structure, indicating a comparable content of sp^3^ and sp^2^ components.

The main differences noticed in the case of Au/ND is the enhancement of bands at 1070 cm^−1^ and the reduction of the band at 1630 cm^−1^, which are related to oxygenated functional groups conjugated or not conjugated with sp^2^ C respectively. In particular, the lowering of the signal at 1630 cm^−1^ can be assigned to the OH groups, while the emergence of a band at 1070 cm^−1^ can be assigned to the stretching of the C-O bond. Both features strongly suggest that the hydroxyl groups at the nanodiamond surface play a major role in the Au^+3^reduction process.

**Figure 2:**
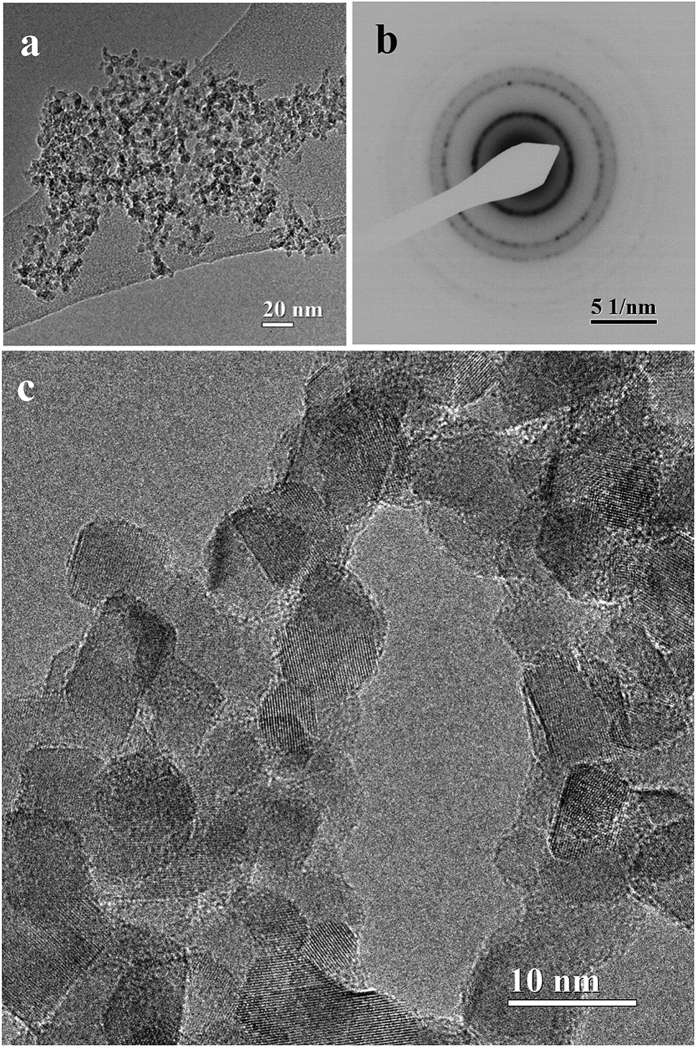
Evidence of ND crystallites from TEM. (**a**) Bright field TEM image of nanodiamonds aggregates with a grape-like shape and (**b**) The corresponding electron diffraction pattern. (**c**) High resolution image of ND polyhedral particles.

TEM bright field image of a purified ND sample (Fig. 2a) shows the typical organization of ND particles aggregated in a grape-like structure. The corresponding electron diffraction pattern (EDP) is displayed in Fig. 2b.

*d-*spacings measured from the diffraction rings indicate that ND is present in the expected cubic phase (space group *Fd3m*) (see Supplementary Table S1). Besides diamond, no further reflections are present in the EDP, excluding a detectable presence of graphite, which can be identified based on different *d-*spacings. In particular, even the strongest diffraction graphite reflection (corresponding to the interplanar distance *d*_*0002*_ = 0.3354 nm) is absent (Table S1)^45^. As far as morphology is concerned, the HR-TEM in Fig. 2c displays polyhedral nanocrystals about 5 nm in size, connected in irregular chains, a kind of self-assembled organization strictly dependent on the mechanism of electrostatic interactions acting differently between the ND facets^46^. The visible lattice spacing, in agreement with the EDP, are compatible with the diamond unit cell dimension a = b = c = 0.357 nm (PDF#06-0675). Overall, the high-resolution images confirm the high structural quality of the nanodiamonds powder, their phase purity and the narrow particle size distribution.

**Figure 3:**
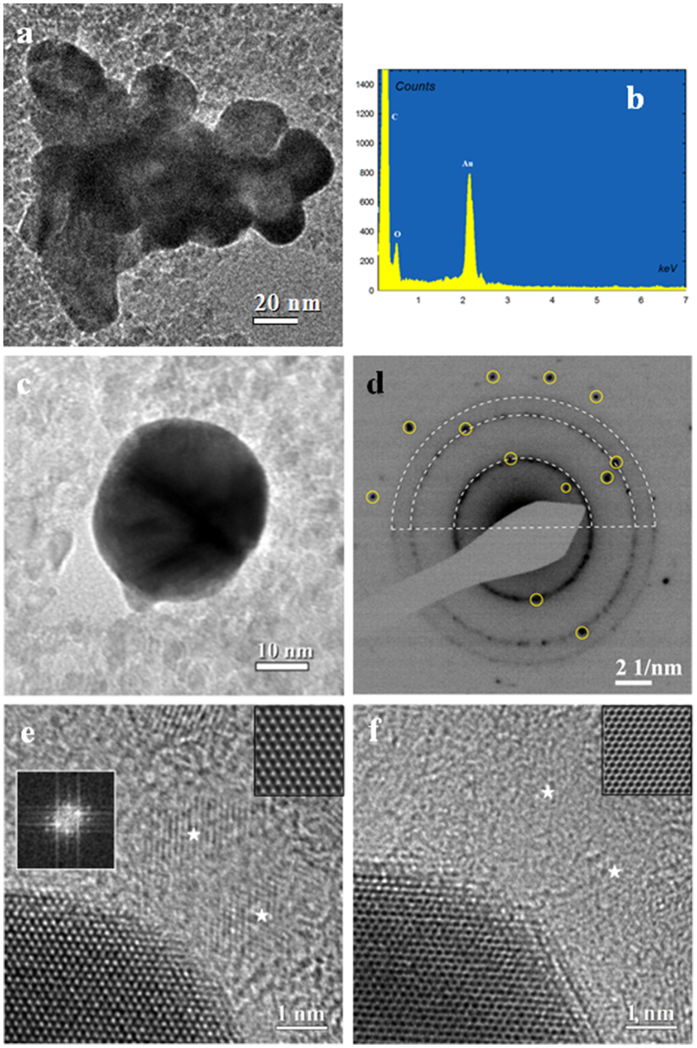
Morphology and structural studies of Au-NDs nanostructures. (**a**) Bright field TEM image of Au-NDs nanoplatelets aggregates. (**b**) EDX spectral image probed on (Fig. 3a). (**c**) Bright field TEM image of single nanoplatelets. (**d**) EDP taken from (Fig. 3c) shows distinct and spotted diffraction rings corresponding to the ND cubic phase (white dot arcs) and diffraction spots belonging to the face-centered-cubic Au phase (yellow circles). (**e**) High resolution TEM image at the edge of an Au nanoplatelet and NDs labeled by white stars. Insets: left side, FFT pattern of Au crystallite of hexagonal shape; right side, simulated HRTEM image of fcc Au along <111> zone axis. (**f**) High resolution TEM image of (Fig. 3e) with different defocus conditions. Inset: simulated HR-TEM image of fcc Au along <111> zone axis.

Preliminary morphological observation by bright field TEM of the Au/ND particles collected after the Au reduction evidences the presence of typical nanoparticle aggregate, where single nanoparticles are larger than observed for pristine ND powder as reported in Fig. 3a. EDX spectroscopy, used to ascertain the particle chemical composition, evidences the presence of two intense peaks (Fig. 3b) belonging to C and Au, confirming that the particles are aggregates constituted by diamond and gold, while the weak oxygen peak can be ascribed to the large presence of O-H groups at the nano-diamonds surface. The lack of any other contribution highlights the high purity of the Au/ND aggregates.

In conclusion, the chemical analysis by EDX supports the high quality of the synthetic protocol able to produce hybrid aggregates, free, within the analytical sensitivity, of salts and/or of unreacted species, used in the various steps of the procedure.

A closer inspection on single non-aggregated particle evidences a typical quasi-ellipsoidal shape of about 30 nm in size (Fig. 3c). Electron diffraction performed on similar entities allows establishing with high precision the crystallographic features. The typical EDP, reported in Fig. 3d, shows the presence of two superimposition different diffraction patterns. In fact the diffraction rings can be indexed according the structure of diamond with space group *Fd3m,* (strong rings marked as white arcs), while the single diffraction spots (evidenced by yellow circles) belong to the face-centered-cubic (fcc) Au with a space group 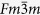.

The EDP details reveal so a particular aggregation of the Au and ND phases. In fact, the structure of the EDP displays an intermediate situation between a cluster of randomly oriented Au nanoparticles characterized by typical continuous diffraction rings, and the high intensity spot pattern of a single crystal Au. The presence of a few distinct diffraction spots of low intensity belonging to the Au structure is typical of a structure where a limited number of Au single nano-crystal are present. A more careful analysis reveals that only one weak spot belonging to the (111) Au reflections can be observed, and its second order (222) reflection plane is absent, despite the high intensity expected for this family of reflections. This can be caused by preferred orientation of the Au nanoplatelets having the [111] crystal direction almost parallel to the electron beam. This finding is confirmed by the high resolution images, which FFTs evidences a preferential [111] zone axis orientation.

To get more detailed information on the microstructure and on the assembling of Au nanoplatelets and NDs, it was necessary to combine HR-TEM, FFT, IFFT, and HR-TEM-EDP simulations.

The HR-TEM image reported in Fig. 3e shows the edge of a gold nanoplatelet characterized by a hexagonal lattice with a spacing *d* = 0.144 nm. This symmetry indicates a [111] zone axis, as confirmed by the FFT shown in the inset. In addition, isolated ND nanograins, with lattice spacing *d* = 0.126 nm, can be observed in the same image (marked with white stars).

The thickness of the Au/ND nanoplatelets was evaluated by comparison of experimental and simulated HR-TEM. To this purpose, a second HR-TEM image (Fig. 3f) was recorded on the same region by changing the focusing conditions. In these experimental conditions a full contrast inversion is observed which is coherent with a sample thickness of about 10 nm and a defocus difference of about 6 nm. The simulated HR-TEM images of fcc Au along <111> zone axis are displayed as top-right insets in Fig. 3e,f (simulations procedures are reported in SI). The evaluated particle thickness shows that the size of ND (4–5 nm) is smaller than the value derived for the Au crystallites. Summarizing the results reported in Fig. 3 it is possible to deduce that Au/ND composite particles can be described as quasi-ellipsoidal nanoplatelets having a diameter of about 30 nm and a thickness of 10 nm. From the EDP in Fig. 3d, the number of incorporated ND particles can be estimated roughly in the range of 10–20 units.

Further analysis has been carried out in order to deeper investigate the mutual organization of Au and ND nanograins in the produced nanostructures.

**Figure 4:**
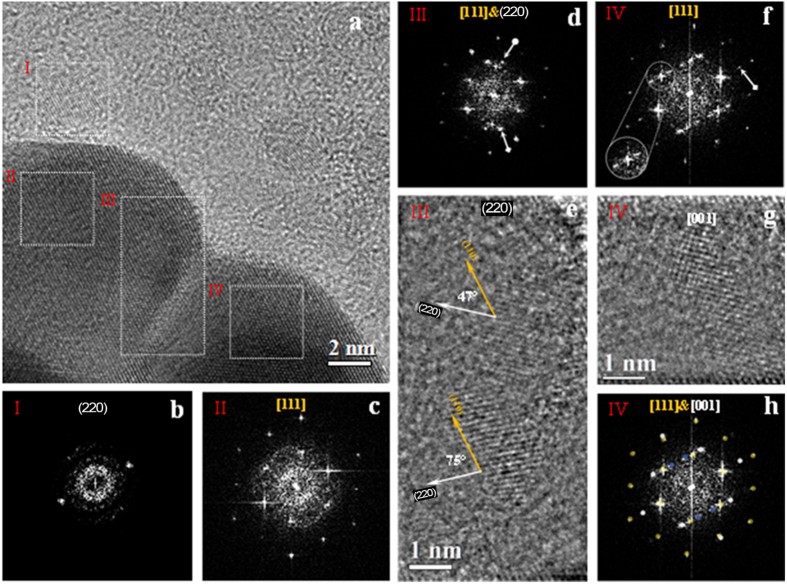
Evidence of Au-ND nanocrystals from High resolution TEM. (**a**) Bright field HRTEM image of Au-NDs edge. (**b**) FFT pattern of region I shows the (220) plane of one ND. (**c**) FFT pattern of region II shows Au atomic layers oriented along [111] axis. (**d**) FFT pattern of region III shows Au hexagonal array and twosome internal symmetric reflections belongs to the ND (220) plane. (**e**) IFFT image of (Fig. 4d) without Au contribution. (**f**) FFT pattern of region IV. (**g**) Identification of the FFT diffraction spots of (Fig. 4f) by electron diffraction simulation. The Au cubic phase orientated along [111] zone axis is marked by yellow spots, the silver spots corresponds to the ND cubic phase orientated along [001] zone axis, and gold defect are marked by blues spots. (**h**) IFFT image of (Fig. 4f) without gold contribution.

The HR-TEM image reported in Fig. 4a shows the edges of two Au/ND nanoplatelets, recorded from the aggregate displayed Fig. 3a, connected by a defect-free buffer region forming a crystalline visible area of about 170 nm^2^. In this region we observe a continuity of Au (110) planes, as shown also by the FFT reported in Fig. 3d. The observed defect-free region can be related to an isotropic growth capable to merge two single Au nanoplatelets into a highly ordered thin nano-object.

To the best of our knowledge, a full understanding of the growth mechanism of Au nanoparticles is still a challenging task, although some conclusions have been already reached. As an example, it has been found that the presence of a single twin plane in the seed leads to the formation of triangular prisms, whereas in the presence of two parallel twin planes hexagonal nanoparticles are formed^28^,^47^. Defects and grain boundaries play a critical role in the formation of different shapes of nanoparticles. In particular, the grain boundaries allow the coexistence of adjacent crystallites with different orientations within the same nanoparticle, e.g., an Au decahedron nanoparticle is constituted by fivefold grain boundaries^26^. A further structural parameter that influences the atomic arrangement of nanoparticles with triangular or hexagonal shape is the thickness of the fcc layers, which, in turn, defines the size of the crystal facets on each edge^47^. In our case, the atomic scale experimental observations have ascertained that the composite Au nanoparticles are thin nanoplatelets with quasi-ellipsoidal shape and without grain boundaries. This last feature is quite unexpected on the basis of literature data, relative not only to Au. In fact, to the best of our knowledge, only circular Ag nanoplatelets similar to our sample have been proposed by Jiang *et al*.^48^, who reported information about the diameter (10 nm) and the atomic crystal orientation (111).

To get further details on the structure, the image analysis through the sequence of FFTs, IFFTs and their elaboration, has been used owing to the capability of fast and reliable discrimination^49^,^50^,^51^ of the presence of an individual nanodiamond crystallite embedded in the Au based particles. Alternative approaches like Multislice Simulation of a high-resolution TEM image or 3D electron tomography are possible but, undoubtedly, more time consuming and complex.

A more detailed analysis of the HR-TEM image shown in Fig. 4a has been performed by FFT on selected image areas. In fact, HR-TEM is based on a phase-contrast imaging mechanism, which is not directly related to the present atomic species. Moreover, the complete identification of ND crystals embedded into the Au/ND hybrid nanomaterial is complicated by the difference in the atomic number between the two phases and in particular by the light nature of the C atoms constituting the ND. In fact, the identification of light phases into a heavy matrix is quite difficult owing to a low intrinsic contrast, a non-straightforward imaging mechanism and the presence of aberration in the microscope optical system causing artifacts in the images.

On the other hand, even if Au and diamond have similar atomic organization in a cubic phase, they can be distinguished by the different bond length and so by the unit cell lattice parameter, so that electron diffraction and related techniques represent reliable tools for the structural analysis and phase identification. In order to ascertain the presence of different phases by enhancing the contribution deriving from small nano-crystals embedded in the structure, the FFT analysis has been performed on selected image nano-areas. This method, which is analogous to nanodiffraction without the limitations imposed by the microscope aberrations or by the beam convergence, can provide easily structural information and mutual crystallographic orientation. Four representative regions enclosed by white boxes and labeled by roman numbers from I to IV (Fig. 4a) have been selected, and the corresponding FFT patterns are reported in Fig. 4b–d,f), respectively.

The FFT reported in Fig. 4b,c, related to regions I and II, evidence the nano-diffraction features of the ND (Fig. 4b) and of Au (Fig. 4c) crystallites, respectively. The two symmetric reflections shown in Fig. 4b (region I), belonging to the diamond cubic phase, have been indexed as (220) planes. The hexagonal pattern of Fig. 4c (region II) has been associated with [111] zone axis of Au nanoplatelets. This result further supports the findings of EDP analysis (Fig. 3d), where the diffraction spots relative to the (111) crystallographic plane were absent.

The indexing of the present diffraction spots in Fig. 4d, where the FFT of region III is reported, evidences, also in this case, a superimposition of the ND and Au patterns. In fact, in Fig. 4d, besides Au spots, two symmetrical reflections with different intensities, marked by white arrows, belonging to two ND crystallites with the same lattice spacing *d*_*220*_ = 0.126 nm can be observed. In order to highlight the nanodiamond structure, the contribution of the Au crystal to the FFT has been subtracted before performing the inverse FFT (IFFT), recovering so the image in the real space. This procedure evidences the presence of two separate nanodiamond crystallites (Fig. 4e), having a structures with the same crystalline features already observed in region I (see Fig. 4b).

The different intensity of the lattice fringes of the ND can be related to different height indicates that the focal plane imaging intersects their atomic layers at different heights along the axis normal to the image plane. Since the lattice fringes are visible for both the separated NDs, we can deduce that the maximum defocus value containing the carbon lattice planes cannot exceed 4 nm based on the known thickness of the ND. On the basis of this qualitative structural information, we can assume that one or both ND crystallites are located within the Au atomic layers of 10 nm estimated thickness, which is larger than the ND thickness (about 4 nm).

Simple geometrical measurements have been performed on the ND lattice fringes of region III (Fig. 4e). The (220) planes of the ND lattice fringes (white arrows) form angles of about 47° and 75° with respect to the (110) gold lattice fringes (yellow arrow, corresponding to about 3π/12 and 5π/12 rad). These rotation angles have been associated to the interaction interface between the Au and ND atomic layers. Since both Au and ND crystals are characterized by the same cubic structure, we can estimate approximately all possible rotation angles as [110]Au^[110]ND = π/3 ± n**·**π/12 (n = 1, 3, 5…), considering van der Waals interactions at the interface. On the basis of these qualitative observations, we can assert that the measured angles among the Au/ND (220) planes are iso-orientated. Additional information supporting our considerations is included in the supplemental material (see in particular Fig. 2S).

A different pattern for ND/Au structure is displayed by the FFT of region IV (Fig. 4f) where, besides the hexagonal array of the Au lattice, a further extra array of diffraction spots can be noticed (white arrow). The inset shows in detail one of the Au (110) spot splitting along the [111] zone axis (white circle). To identify the extra array of diffraction spots, we used the same approach applied to the FFT of region III in order to reveal any ND hidden among the gold atomic layers. The IFFT shown in Fig. 4g evidences ordered lattice fringes with the same *d*-spacing previously determined for ND. In order to accurately determine the crystallographic features of Fig. 4f, the FFT nano-diffraction pattern taken from region IV has been processed by electron diffraction simulation. The simulated EDP that better overlaps the experimental EDP of Fig. 4h is the one corresponding to the [001] zone axis of the ND cubic phase (white circles), in which the calculated EDP has been referred to the [111] Au phase (yellow circles). Unfortunately, the *d*-spacing of the inner four reflections (blue circles) cannot be precisely and unambiguously measured because of the poor electron transmittance and of the slight distortions of the FFT pattern arising from the residual aberrations. Since these extra inner spots have a *d*-spacing that we estimated to be slightly larger than the highest one of standard Au (*d* = 0.236 nm), their existences could be correlated to the existence of longer interatomic distances induced by the presence of carbon specie, as reported in literature^52^.

To have a better statistics on our results concerning the structure of Au/ND particles and to highlight further peculiarities, we report here the analysis performed on a different portion of the same cluster of Fig. 3a.

**Figure 5:**
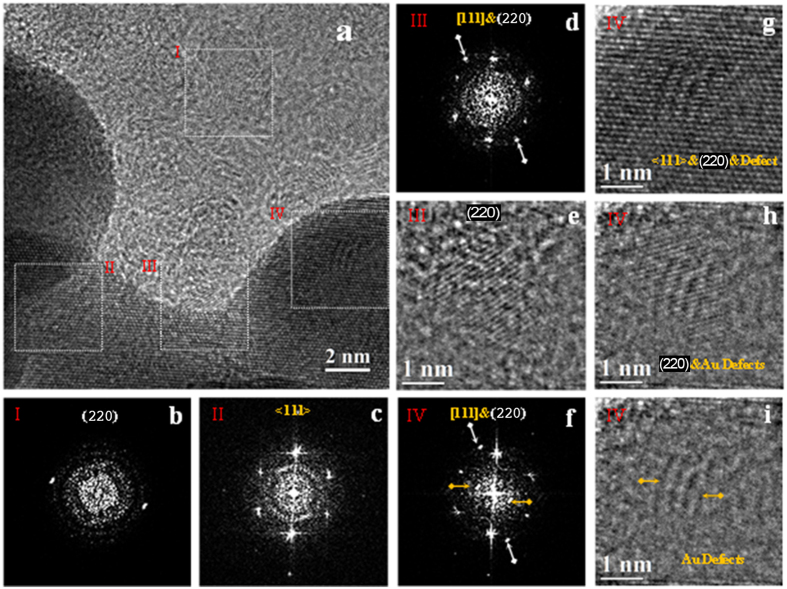
Evidence of Au-NDs nanocrystals from High resolution TEM. (**a**) Bright field HRTEM image of Au-NDs edge. (**b**) FFT pattern of region I shows the (220) plane of one ND. (**c**) FFT pattern of region II shows Au atomic layers oriented along [111] axis. (**d**) FFT pattern of region III. (**e**) IFFT image of (Fig. 5d) without gold contribution showing the pure ND on the gold edge crystallite. (**f**) FFT pattern of region IV. (**g**) IFFT image of (Fig. 5f). (**h**) IFFT image of (Fig. 5f) without gold contribution. (**i**) IFFT image of (Fig. 5f) without gold-nanodiamond contribution.

The image in Fig. 5a, shows partially overlapped Au nanoplatelets and ND crystallites. Image analysis by localized FFT in selected areas, shows Au atomic layers with hexagonal [111] zone axis simmetry. The FFT analysis of regions I (Fig. 5b) and II (Fig. 5c) shows the contemporaneous presence of ND and Au, assessing a structure composed by Au grown at the surface of ND.

Region III (Fig. 5d) exhibits a superposition of Au and ND FFT patterns. By the already described procedure, a nanodiamond grain with [110] zone axis orientation attached to the edge of an Au nanoplatelet can be observed. The same procedure has been able to reveal, also in region IV, the presence of ND crystallites. In detail, the FFT analysis (Fig. 5f) shows several diffraction spots. Besides the hexagonal FFT pattern of the Au crystallite, two external spots belonging to a single ND crystallite (*d* = 0.126 nm, white arrows), and again we have two inner extra spots (denoted by yellows arrows) with interplanar distance *d* = 0.236 nm, that we estimated to be slightly larger than the highest one of standard Au. The IFFT performed on region IV (Fig. 5g) shows irregular lattice fringes overlapping the regular one. With the aim of elucidating the contribution of the present phases, several additional IFFT images have been reconstructed by subtracting different contribution to the FFT of Fig. 5f. The IFFT image of Fig. 5h, obtained by subtracting only the contribution of the [111] Au layers, clearly shows the presence of a single ND overlapping four broad anomalous fingers. The last IFFT image (Fig. 5i), obtained by subtracting also the ND FFT signal, reveals the presence of four broad lattice fringes. This occurrence can be probably related to some defect formed during the Au growth. All the defect-free Au atomic layers observed in the analysed area of about 756 nm^2^ are orientated along the [111] crystallographic direction. This is in agreement with literature data which report that Au [111] surface has the lowest energy, thus favoring strong atomic bonding during the growth^53^. Such finding can be likely correlated to the growth mechanisms of the Au nanoparticles interacting with nanodiamonds with the same cubic phase symmetry.

In conclusion, the HR-TEM observations evidence that the Au nanoplatelets have the same atomic orientation and are terminated with a (111) plane, as also confirm the SAED results of Fig. 3d. Finally, it is interesting to notice that also nanodiamonds entrapped into the Au nanoplatelets or attached to the surface show the same crystallographic orientation corresponding to a [001] zone axis (Fig. 4g,h).

## Conclusions

Au/ND assemblies obtained by means of an innovative synthesis route have been carefully investigated in order to elucidate their structure and morphology at the nanoscale. The characterization protocol developed in the present work could be also adapted to the analysis of other two-phase nanocrystalline systems.

Hybrid Au/ND nanostructures have been produced by reducing an Au(III) salt in presence of aqueous dispersion of OH functionalized nanodiamonds, without any further reducing agent or heat treatment.

The reduction of the Au ions occurs through redox reactions induced by the OH groups present on the ND surfaces. The amount of reduction sites depends by the ND treatments and by their efficiency in functionalizing the ND surfaces with OH groups (in form of both enolic or conjugated alcohol).

A proof of the role played by the OH groups in the reduction of Au(III) ions is provided by the comparison of the Raman spectra taken before and after the precipitation reaction. These spectra also indicate that the amount of the OH groups was appropriate for the gold reduction.

The synthetic approach utilized in the present work presents some interesting aspects, here briefly summarized. First, the preparation procedure is coherent with a “sustainable chemistry” approach even if the key point is represented by the capability of this novel procedure to create unusual structural arrangement of the Au and ND crystallites. The Au/ND organization in our samples is indeed very different from what obtained by conventional methods used for the decoration of nanoparticles with Au. This is ascribed to the peculiar capabilities of ND surfaces, which are able to engineer the synthesis pathway by controlling the growth of the reaction partners, by mediating the crystallization processes, by modulating the size and shape tailoring the architectures of the hybrid nanomaterials.

However, in order to master the synthesis of materials at the nanometer scale, a deep knowledge of the fundamental aggregation mechanisms is required while the use of high spatial resolution tools allows establishing how the properties of the synthesized matter are size- and shape-dependent.

In this respect, the approach used here to investigate the organization of the Au/ND aggregates, represent a powerful tool to investigate directly the assembling mechanism, and can be considered as a suitable protocol for the analysis of similar hybrid structures based on the arrangement of different nanocrystalline species.

Image analysis and HR-TEM observations revealed the structural details of both Au and ND crystallites and provided useful information on their mutual organization. Local analysis of HR-TEM images evidenced the presence of crystalline Au nanoplatelets with fcc symmetry and oriented along the [111] zone axis, grown with defined orientation relationship on ND surfaces. It has been ascertained that, under the conditions of the present experiments, Au particles have a quasi-ellipsoidal shape with average width of about 30 nm and average height of about 10 nm; the ND grains are embedded into the 2D Au particles.

Such unusual assembling suggests the presence of a cooperative mechanism between Au and ND, likely based on interfacial interactions finally leading to hybrid Au/ND nanomaterials.

Even if gold and diamond belong to different crystal space groups, they share a common atomic cubic organization so that a common [111] crystallographic orientation, can support interface interactions and mutual orientation relationship.

The growth of Au nanoparticles into ellipsoidal shapes and the lack of crystal defects like the multiply twinning commonly observed on Au nanoparticles^25^,^54^, or grain boundaries, are features which can be ascribed to the peculiar properties of the ND surfaces. In this context, the NDs act as sinks for the defects of the Au nanoparticles.

The absence of defects makes these Au-based systems particularly suited for optical and photonic applications. Moreover, the Au/ND aggregates here studied can be considered not inherently toxic^55^, owing to a preparation protocol in aqueous media without reducing agents, additives or stabilizers. Moreover, whereas Au nanoparticles of 1–2 nm in size have been recognized to be potentially toxic for their irreversible binding to cells, in the case of a size larger than 15 nm the effect is not observed^56^. This last characteristic is a key point for bio-related applications such as imaging and labeling of biological tissues.

Summarizing, the hybrid Au/ND nanostructures produced in the frame of the present research display high scientific interest for the study of cooperative mechanisms leading to novel crystallization pathways and represent a model system for the settling of an exhaustive characterization protocol.

On the other hand, the material itself, due to the easy preparation and its high reproducibility, represents an innovative concept for efficient, stable and biocompatible optical, photonic and electronic systems. Hybrid Au/ND systems can be also excellent field emitters^57^, and represent a viable solution for next generation cathode materials for high-brightness FE and plasma-based display devices.

In our labs we are applying such exciting materials for the assembling of sensors and biosensors platforms and as novel nanosources for light amplification.

## Additional Information

**How to cite this article**: Matassa, R. *et al*. Structural and morphological peculiarities of hybrid Au/nanodiamond engineered nanostructures. *Sci. Rep.* **6**, 31163; doi: 10.1038/srep31163 (2016).

## Supplementary Material

Supplementary Information
