# Supplementary material for: Structural and morphological peculiarities of hybrid Au/nanodiamond engineered nanostructures
Source: Sci Rep. 2016 Aug 12;6:31163. doi: 10.1038/srep31163 (PMC4981890; doi:10.1038/srep31163)
Supplement: Supplementary Information [file srep31163-s1.pdf]

Structural and morphological peculiarities of hybrid Au/nanodiamond engineered nanostructures

SUPPORTING INFORMATION

Roberto Matassa,[†] *Silvia Orlanducci,*[‡] *Giacomo Reina,*[‡] *Maria Cristina Cassani,*[§] *Daniele Passeri*[†], *Maria Letizia Terranova,*[‡] *and Marco Rossi**[,†,ǁ]

[†]Department of Basic and Applied Sciences for Engineering, Sapienza University of Rome, Via Antonio Scarpa 16, 00161 Rome, Italy.

[‡] Department of Chemical Science and Technology-MINIMAlab, University of Rome "Tor Vergata", Via della Ricerca Scientifica, 00133 Rome, Italy.

[§] Department of Industrial Chemistry 'Toso Montanari', University of Bologna, Viale del Risorgimento 4, 40136 Bologna, Italy.

[ǁ]Center for Nanotechnology for Engineering (CNIS), Sapienza University of Rome, P. le A. Moro 5, 00185 Rome, Italy.

Supporting Information.

Table S1. Interplanar spacing (nm) of observed reflections for Au-ND nanocomposite of figure 2b and 3d with d_{111} of NDIA as standard calibration. Miller indexes and corresponding reference data of Au (card PDF#00-002-1095), ND (card PDF#0675 and reference [A]), and Graphite (card PDF 41-1487).

N_{Rings}	Measured	hkl	Gold	Diamond		hkl	Graphite
1	0.2362	111	0.236	–		002	3.354
2	0.2061	111	–	0.206		100	2.131
3	0.2044	200	0.204	–		101	2.031
4	0.1740	200	–	–		102	1.798
5	0.1450	220	0.144	–		004	1.677
6	0.1261	220	–	0.126		103	1.542
7	0.1231	311	0.123	–		110	1.230
8	0.1174	222	0.117	–		112	1.155
9	0.1086	311	–	0.108		105	1.135
10	0.1036	222	0.102	–		006	1.118
11	0.0932	331	0.093	–			
12	0.0916	420	0.091	–			
13	0.0893	400	–	0.089			
14	0.0834	422	0.083	–			
15	0.0823	313	–	0.082			
16	0.0802	204	–	–			
17	0.0784	511	0.078	–			
18	0.7350	224	–	0.073			

High resolution transmission electron microscopy simulations

The multislice method was employed to simulate HREM image of gold nanoparticle. The simulations conditions were referred to the HR-TEM instrument (JEOL JEM-3000F FEG TEM): acceleration voltage 300 kV, spherical aberration of C_s =0.6 mm, three-fold astigmatism of 850 nm, beam divergence of 0.10 mrad and focal spread of 5 nm.

Multislice simulations were conducted for a 15 nm thick gold crystal in [111] zone axis of a supercell of 5x5 unit cells. Defocus series were calculated with an objective aperture of 12.2 mrad setting 2.0 nm defocus step, being the starting defocus around -41.0 nm according to the Scherzer focus parameter of the TEM.

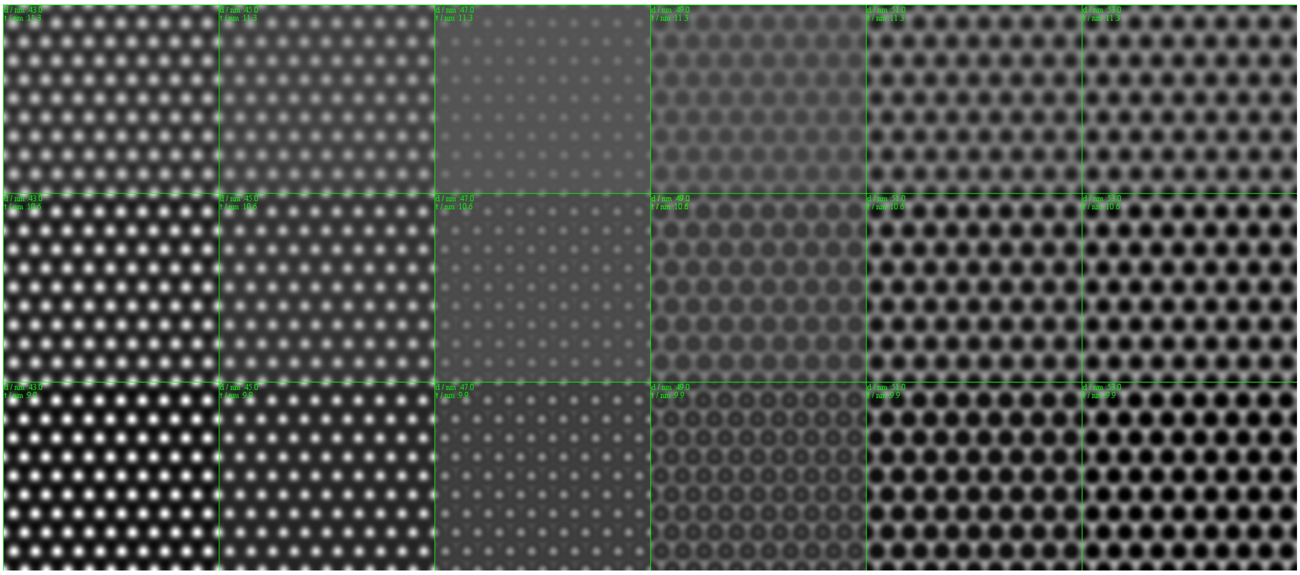

Figure 1S. HREM defocus-thickness map image of gold atomic layer.

[A] M. L. Terranova, D. Manno, M. Rossi, A. Serra, E. Filippo, S. Orlanducci, and E. Tamburri, *Cry. Growth Des.*, **2009**, *9*, 1245-1249.

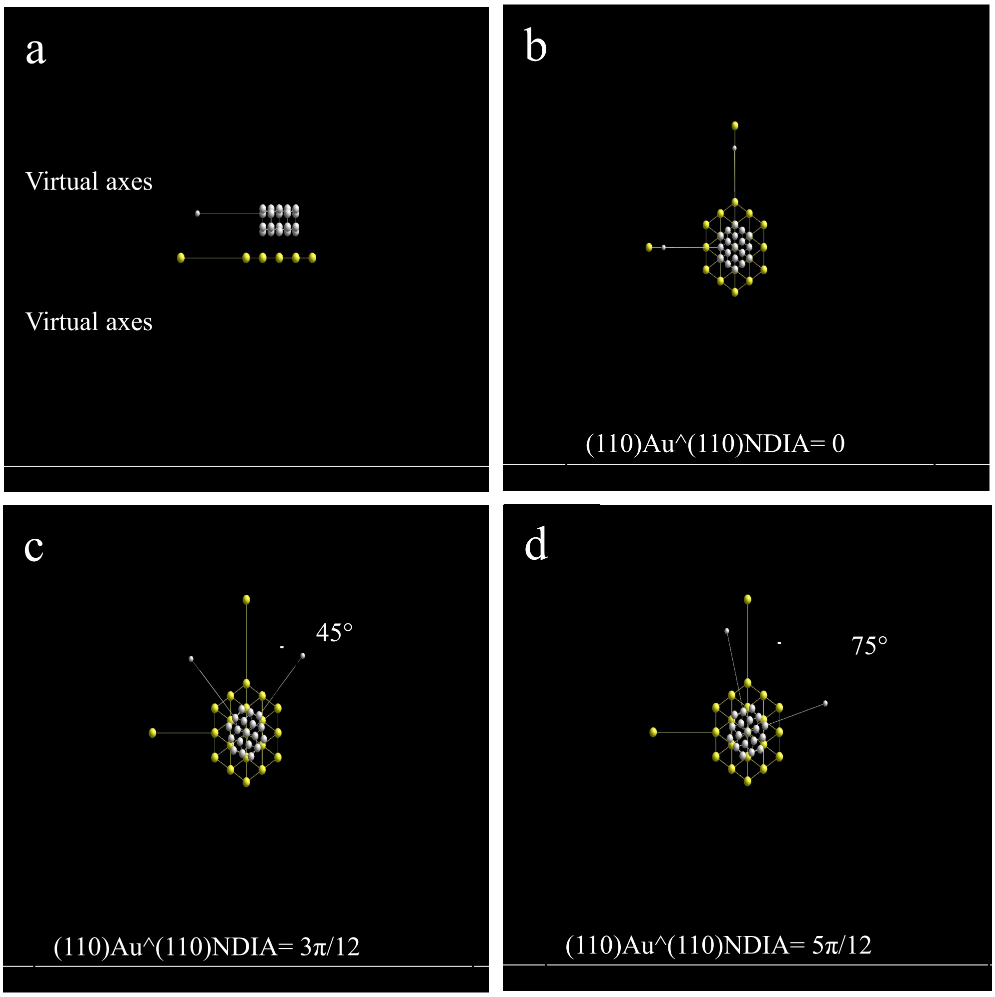

Figure 2S. Atomic sketch views of two NDIA layers (white balls) stacked on one gold layer (yellow balls) both oriented along the [111] zone axis. a) Au and NDIA layers separated by non-covalent bonding (Van der Waals interactions). b) (110)Au^(110)NDIA= 0 radian. c) (110)Au^(110)NDIA= 3 /12 radians. d) (110)Au^(110)NDIA= 5 /12 radians.
